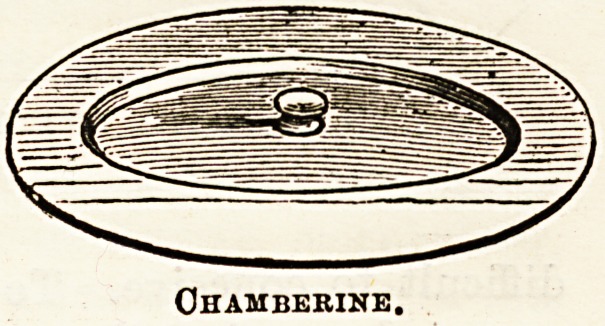# Practical Departments

**Published:** 1896-02-01

**Authors:** 


### PRACTICAL DEPARTMENTS.

### UNBREAKABLE WARE.

The convenience of being able to substitute unbreakable for earthen and china ware without sacrificing all the advantages of the latter is very great, perhaps particularly in institutions where smashings are unavoidably frequent and the expense incurred consequently considerable. The difficulty has been to manufacture a material which will stand water and the action of soap, acids, &c., but this seems to have been satisfactorily overcome by the Unbreakable Pulp Ware Company (Limited), of Thetford, in Norfolk, who have succeeded in producing a substance which will stand hard wear and tear, is light and cleanly, and will not chip or break. In its rough state, after passing through the first processes of grinding, moulding, and pressing, it has a soft surface, becoming at last hard and solid by being put through hydraulic stamping machines of great power. The varnishing and surfacing finally give an enamelled article, which it is claimed has been proved to stand hot-water for years. That this is the case is testified by the fact that jugs and basins and water pails have been and are supplied in large quantities to the Government for use in her Majesty's prisons.

Nursery Tub.

The first of the accompanying illustrations shows a "tub" for nursery use, and for the purpose of a baby's bath. Besides the qualities of light weight and durability, it is an advantage that the chilly feeling of earthenware or zinc is absent. Basins and tubs can be had in all sizes, and these basins are excellent for washing glass and china in, saving breakages by reason of the softness of the ware. All toilet appliances, jugs and basins, soap-dishes and water-carriers are made in this ware.

Especially to be recommended are the excellent little covers for bed-room utensils, of which mention has before been made in The Hospital. They are eminently sanitary and non-absorbent, and every house should possess a supply. They are inexpensive enough—10s. 6d. a dozen. Ten dozen have just been despatched to the Kimberley Hospital, South Africa.

Besides these useful articles for the nursing and the sick-room, the show-rooms of Messrs. W. B. Fordham and Sons (Limited), 36, York Road, King's Cross, the distributing

Water Carrier.

Chamberine.

agents of the company, contain samples of a number of flower-pots, trays and waiters, mugs, and other things in ware of various colours, some of which are prettily decorated; and this decorative work has an industrial value of its own, for a large number of women are employed at the Thetford works in this department, transferring and executing designs. The price in all cases is extremely moderate, and we fancy the pulp ware will only need to be known to become generally popular.

## Figures and Tables

**Figure f1:**
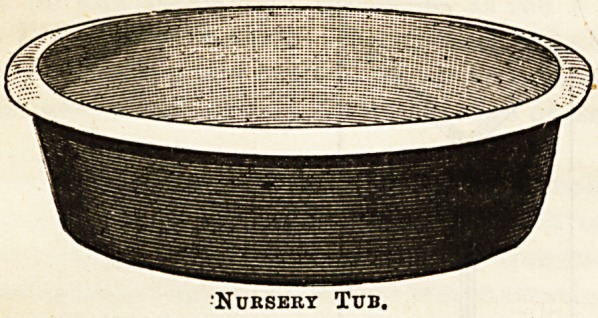


**Figure f2:**
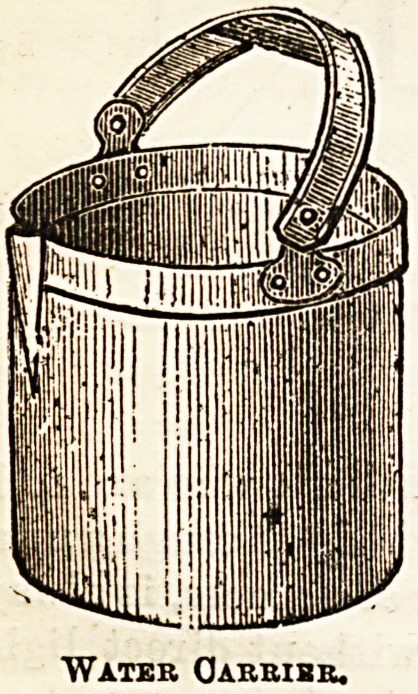


**Figure f3:**